# Quantitative Spatial Analysis on Radiographic Features of Rotator Cuff Calcifications: An Exploratory Study

**DOI:** 10.3390/biomedicines13030551

**Published:** 2025-02-21

**Authors:** Ju-Hyeon Kim, Dahae Yang, Jae-Hyun Lee

**Affiliations:** 1 Department of Physical Medicine and Rehabilitation, Kosin University Gospel Hospital, Busan 49267, Republic of Korea; virtuouswide@naver.com; 2 Department of Laboratory Medicine, Kosin University Gospel Hospital, Busan 49267, Republic of Korea; laluna11@naver.com; 3 Department of Laboratory Medicine, Kosin University College of Medicine, Busan 49267, Republic of Korea; 4 Department of Physical Medicine and Rehabilitation, Pusan National University Hospital, 179 Gudeok-ro, Seo-gu, Busan 49241, Republic of Korea; 5 Department of Artificial Intelligence Convergence, Pukyong National University, Busan 48513, Republic of Korea

**Keywords:** calcific tendinitis, radiography, rotator cuff tendon, shoulder, heterogeneity

## Abstract

**Background/Objectives**: Plain radiography is the primary diagnostic tool for calcific tendinitis of the shoulder. Several qualitative grading methods have been proposed to represent the pathophysiologic phase and guide treatment decisions. However, these methods have demonstrated low reliability, complicating their effectiveness for such purposes. This study aims to perform the first quantitative analysis of calcific lesions using radiographic imaging and explore their correlation with ultrasonographic parameters to enhance their diagnostic utility. **Methods**: A total of 57 shoulders presenting with painful calcific tendinitis in either the supraspinatus or subscapularis tendon were reviewed. The calcific deposits and tendon regions of interest were meticulously identified and annotated. Image brightness was reduced to 256 grayscale levels, and descriptive and heterogeneity parameters, including skewness, kurtosis, complexity, and entropy, were quantified and analyzed. **Results**: In the region of calcification, the average grayscale values were 21.69 units higher than those of tendon tissue. All spatial heterogeneity parameters, except for skewness, demonstrated statistically significant differences when compared with the adjacent tendon. Notably, entropy and complexity were the most distinctive features, with an area under the curve of 0.93 and cut-off values of 4.62 and 4.18, respectively. Significant correlations were observed between the heterogeneity parameters and ultrasonographic findings, such as bursal contact and peri-calcific hypoattenuation. **Conclusions**: Calcific deposits demonstrated not only increased brightness in grayscale levels but also distinct spatial heterogeneity. The correlation with ultrasonographic findings indicates that these heterogeneity parameters may reflect underlying pathophysiological characteristics. Future prospective research could explore the whole temporal changes of calcifications more thoroughly.

## 1. Introduction

Diagnosing and treating shoulder calcific tendinitis is of utmost importance, as it is prevalent and frequently induces severe pain [1,2]. This condition arises from the accumulation of poorly crystalline carbonated apatite [1,3], with the shoulder joint being the most commonly affected. Among the structures around the shoulder joint, the supraspinatus tendon is reported to be the most commonly affected, followed by the infraspinatus tendon and subscapularis tendon [4,5]. In rare cases, lesions may also involve the biceps anchor or extend into the muscle belly [6].

Calcific deposits undergo a dynamic life cycle that can be categorized into several stages: pre-calcific stage, the formative, resting, and resorptive phase of the calcific stage, and post-calcific stage [7,8]. During the formative phase, the tendons undergo a mineralization, generally occurring asymptomatically and presenting challenges for lavage. Subsequently, in the resorptive phase, patients often experience intense pain due to inflammatory responses marked by the formation of peripheral vascular tissue and the activity of macrophages and multinuclear giant cells that engulf calcific deposits [7]. This phase involves the liquefaction of deposits, soft tissue swelling, and increased pressure within the tendon [1,9]. Consequently, accurately identifying phasic progression is crucial for the effective treatment of calcific deposits.

Despite the availability of other imaging modalities, conventional radiography is the preferred initial modality for identifying calcific deposits and evaluating their location, size, and morphology [1,10]. This preference is attributed to its straightforwardness and its ability to eliminate concerns about superimposed bone abnormalities. However, current radiographic classifications based on deposit morphology or appearance—assessed through visual inspection by physicians—such as inhomogeneity and blurriness have demonstrated low reliability, poor reproducibility [11], and inconsistencies when contrasted with other imaging modalities such as ultrasonography, which is recognized for its accuracy in detecting phasic progression [8,11,12,13].

While previous qualitative classifications have yielded suboptimal results, we hypothesize that the spatial variations of apatite crystals in calcific deposits can be elucidated through quantitative analysis of radiopacities, potentially revealing latent information. In this study, we employed grayscale analysis, a technique demonstrated to effectively differentiate tissue pathology in other disease conditions [14,15,16,17,18].

The aim of this study is to quantitatively assess calcific deposits on radiographs in subjects suffering from painful shoulder rotator cuff calcific tendinitis, utilizing grayscale histogram analysis to identify patterns and derive numerical characteristics. Moreover, this study further explores the correlation between radiographic findings and ultrasonographic parameters to enhance the diagnostic utility of radiographic imaging and deepen pathological interpretation.

## 2. Materials and Methods

### 2.1. Study Population

This study conducted a retrospective analysis of the medical records of patients who sought treatment for shoulder pain at a musculoskeletal clinic and were diagnosed with pain-provoking calcific tendinitis in the rotator cuff tendons between March 2019 and December 2021. We specifically included patients with visible calcific deposits in the supraspinatus or subscapularis tendons, using a 30-degree caudal tilting and axillary views to ensure clear visualization of the tendon footprint and precise delineation of the calcific deposit boundaries. Calcifications in other rotator cuff tendons, such as the infraspinatus and teres minor, were excluded from the study since our imaging protocol could not optimally visualize them. Patients with other coexisting pain-provoking conditions, such as frozen shoulder, rotator cuff tear, or inflammatory arthritis, were excluded from the study. Exclusion was determined through diagnostic codes and the assessment sections of the medical records. Other exclusion criteria encompassed the absence of radiographic imaging, the lack of suitable radiographic views for analysis, or incomplete medical records for parameter assessment. Treating cases of recurrence in the opposite shoulder as separate instances, we reviewed 87 cases from a sample of 61 patients. Initially, cases with concurrent frozen shoulder and rotator cuff tendon tears (*n* = 10) were eliminated based on medical records. It was noted that none of the cases involved inflammatory arthritis. The subsequent exclusion phase relied on radiographic evaluations. We excluded cases that either lacked visible calcifications on shoulder radiographs or did not include 30-degree caudal tilt or axial views (*n* = 8). Additionally, cases were excluded if the calcifications overlapped with bone contours or migrated into the bursa, rendering the margins of the calcifications indiscernible (*n* = 12). Following this review, 57 cases of calcific tendinitis were confirmed for inclusion.

This study was approved by the Institutional Review Board of Kosin University Gospel Hospital and was performed in accordance with the guidelines of the Helsinki Declaration. Informed consent was waived, because the data were deidentified and the study design was retrospective.

### 2.2. ROI Capture for Radiographic Analysis

**Figure 1:**
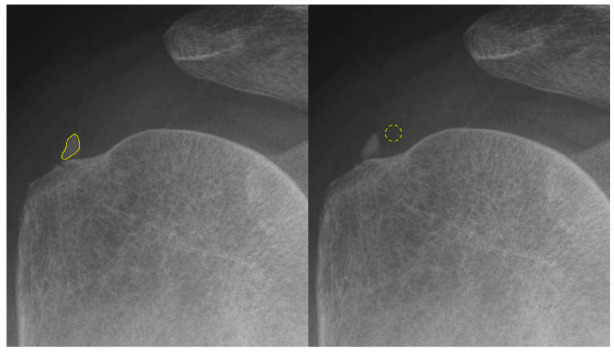
A 59-year-old male patient with calcification on the right supraspinatus. A 30-degree caudal tilting view of right shoulder. Region of interest 1 (yellow outline) was created with a freehand selectionalong the contour of the calcification (**left**). Region of interest 2 (yellow circle) was selected with a radius of 20 pixels at the normal tendon portion right next to the calcification (**right**).

Shoulder X-rays were acquired using the INNOVISION DXII system (DK Medical Systems, Seoul, Republic of Korea), the primary imaging device used at our institution. For the analysis of the supraspinatus tendon, a 30-degree caudal tilt view was employed to optimally visualize the tendon and its footprint on the greater tubercle of the humerus. The subscapularis tendon was assessed using an axial view. To accurately capture both the calcific deposit and the normal tendon areas for grayscale value analysis and quantitative assessment, we employed ImageJ (Version 1.53, National Institutes of Health, Bethesda, MD, USA), an open-access software, to delineate the region of interest (ROI) from the collected raw radiographic images. The original Digital Imaging and Communications in Medicine (DICOM) images, with a pixel spacing of 1.41 × 1.41 mm, obtained from the Picture Archiving and Communication System (PACS) were imported into ImageJ. The pixel brightness intensity values were represented in an 8-bit image. Subsequent grayscale analysis was executed using ImageJ’s default settings, without the application of thresholding or Gaussian blur. Histogram analysis was conducted with 256 bins, and pixel intensities were normalized to a range of 0–255. The ROI was meticulously defined to encompass the calcific deposit area, with careful attention given by a physician. Additionally, a circular ROI was marked on the adjacent tendon tissue located proximal to the calcific deposit, situated between the hypoechoic bursa overlying the tendon and the cortex of the humeral head (Figure 1).

### 2.3. Ultrasonographic Parameters

**Figure 2:**
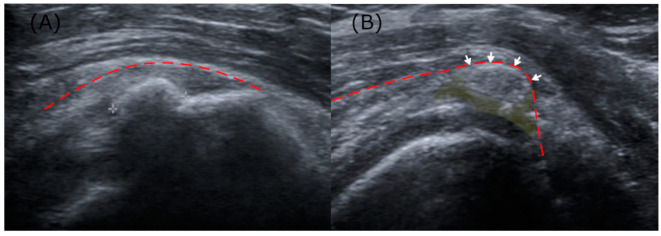
(**A**) An arc-shaped calcification (Sconfienza Grade 1) with acoustic shadowing, lacking both bursal contact and peri-calcific effusion. (**B**) A hypoechoic calcification (Sconfienza Grade 3), where the acoustic shadow is diminished, accompanied by bursal wall contact and peri-calcific tendon effusion. The bursal wall, representing the inferior boundary of the subdeltoid–subacromial bursa, is delineated with a dotted red line; areas where calcific deposits contact the bursa are indicated by arrows; peri-calcific hypoattenuation regions are highlighted in yellow.

Key ultrasonographic parameters encompassed Sconfienza’s classification, shadow faintness, bursal wall contact, and peri-calcific hypoattenuation. Sconfienza’s classification, which grades echogenicity within calcification, was used to grade the deposits as either Grade I (hyperechoic and hard) or Grade II/III (homogeneous and soft/hypoechoic and fluid-like) [5,19]. Shadow faintness was defined as the presence of either partial or complete obscuration of the acoustic shadow. Bursal wall contact was marked by the displacement of the smooth bursal wall by calcific deposits, and peri-calcific hypoattenuation was defined by hypoattenuation of the tendon tissues around the deposits (Figure 2). In the analyses concerning the ultrasound parameters, cases without a shoulder ultrasound performed within a one-month window were excluded. The ultrasonographic parameters were evaluated by a physiatrist with over 10 years of experience in musculoskeletal practice. The evaluations were performed independently, without prior knowledge of the radiographic analysis results.

### 2.4. Statistical Analysis

**Figure 3:**
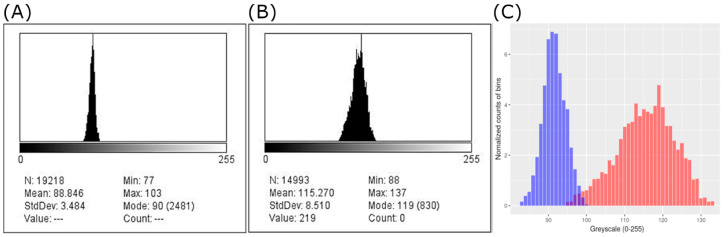
(**A**,**B**) Grayscale histograms generated using ImageJ: (**A**) a normal tendon and (**B**) a calcific deposit. (**C**) The grayscale histograms of standardized regions of interest generated by R Statistical Software, with the normal tendon represented in blue and the calcific deposit in red.

The captured ROIs were converted into grayscale images consisting of 256 levels ranging from 0 to 255. Descriptive parameters, including mean and quartile values, were measured and compared between calcific deposits and normal tendons. Subsequently, histograms were generated (Figure 3), from which several parameters were derived, to represent heterogeneity. In various disciplines, heterogeneity parameters are employed to evaluate diversity within a system or group. Previous studies in the medical field have utilized variables that reflect the internal heterogeneity of soft tissues. In our research, we also employed variables that indicate the heterogeneity of grayscale within ROIs as follows. Skewness and kurtosis were employed to assess asymmetry in the distribution, while entropy and complexity were calculated to evaluate disorder within the distribution. Entropy was defined as the sum of each grayscale’s histogram multiplied by its logarithm base 2, while complexity was defined as E(E − 1)/4, where E represents entropy.

The reliability of manual ROI delineation and derived grayscale parameters of calcifications between two observers was evaluated using the Intraclass Correlation Coefficient (ICC). The Wilcoxon signed-rank test and the receiver operating characteristic (ROC) were employed to investigate the distinction of calcific deposits from adjacent tendon tissues. Associations with ultrasound and other clinical parameters were analyzed using the Mann–Whitney *U* test and Pearson’s chi-square test, adopting a significance threshold of *p* < 0.05. Cohen’s r was used to calculate the effect size. All statistical analyses were conducted on R Statistical Software, version 4.4.0 (Foundation for Statistical Computing, Vienna, Austria).

## 3. Results

The study involved 61 subjects with an average age of 56.35 ± 8.28 years, including 13 male subjects. Among the 57 cases that underwent appropriate shoulder radiography, calcifications were detected in the supraspinatus tendon in 40 instances and in the subscapularis tendon in an additional 17 cases. The demographic characteristics of the two groups are compared in Table 1, showing no significant differences.

The interobserver agreement for all descriptive statistics, including the minimum, maximum, mean, first quartile, median, and third quartile values, demonstrated exceptional reliability, with the ICCs exceeding 0.950. In terms of heterogeneity parameters, entropy and complexity exhibited strong interobserver reliability, whereas skewness and kurtosis showed moderate agreement. The ICC values (with 95% confidence intervals) for these parameters were skewness at 0.831 (0.677–0.915), kurtosis at 0.799 (0.623–0.899), entropy at 0.970 (0.938–0.986), and complexity at 0.971 (0.939–0.986).

### 3.1. Radiopacity

**Figure 4:**
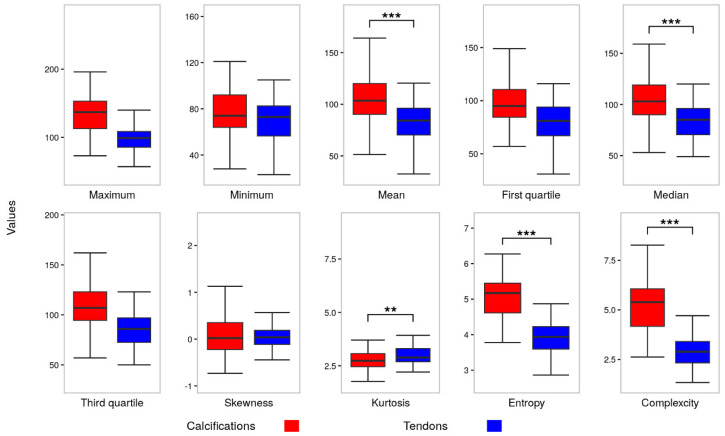
Comparison of the grayscale parameters and spatial heterogeneity between calcifications and tendons. ** *p* < 0.01 and *** *p* < 0.001.

All the descriptive parameters show significantly higher values, except the minimum value. The quantitative radiopacity values are as below. The maximal value was 136.30 ± 31.61, significantly elevated by 36.97 units, in contrast to 99.93 ± 22.37 in the tendon region (*p* < 0.001, effect size r = 0.561). Similarly, the median value was 107.63 ± 24.99, significantly higher by 21.69 units compared to 85.82 ± 21.66 in the tendon region (*p* < 0.001, effect size r = 0.423). The rest of the greyscale values are visualized in Figure 4 as boxplots.

### 3.2. Heterogeneity

The entropy and complexity metrics were notably higher in the calcific region, exhibiting mean values of 5.07 ± 0.61 and 5.26 ± 1.40, respectively, in contrast to 3.94 ± 0.45 and 2.95 ± 0.77 in the tendon regions (*p* < 0.001, effect size r = 0.742 for both metrics). The skewness values were close to zero in both tendon and calcific regions, −0.01 and 0.03 in median value (*p* = 0.869, effect size r = 0.004), while kurtosis displayed 2.71 and 2.89 in median value, respectively (*p* = 0.007, effect size r = 0.232).

**Figure 5:**
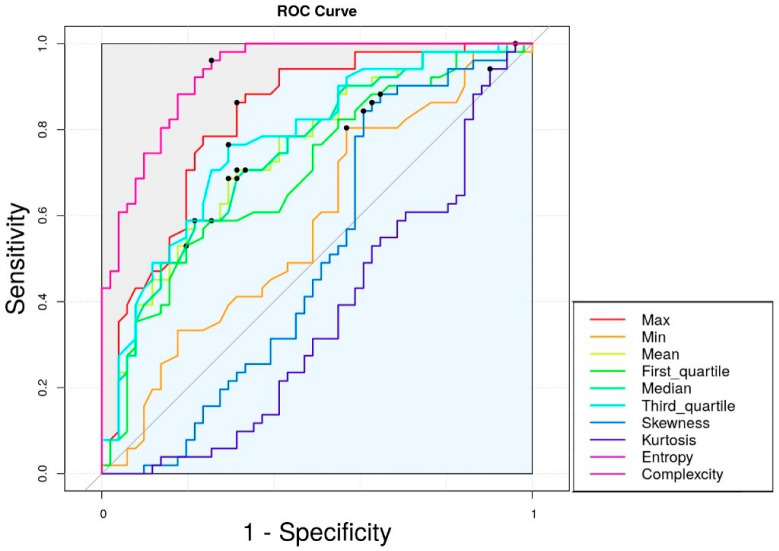
ROC curve of each grayscale parameter. The black dots on each curve represent the cutoff values that optimize sensitivity and specificity. The legend on the right indicates the parameter corresponding to each colored curve.

Table 2 and Figure 5 present the area under the curve (AUC) values of the grayscale parameters. Entropy and complexity demonstrated an AUC of 0.928, with a sensitivity of 84.2% and specificity of 87.7%. The cut-off values were 4.43 for entropy and 3.80 for complexity. In contrast, descriptive parameters such as the mean and first to third quartile grayscale values exhibited poor to acceptable discrimination, with AUCs ranging from 0.594 to 0.777 (*p* < 0.001), except a maximal value of 0.830.

### 3.3. Associations Between Ultrasonographic Parameters and Grayscale Parameters

**Figure 6:**
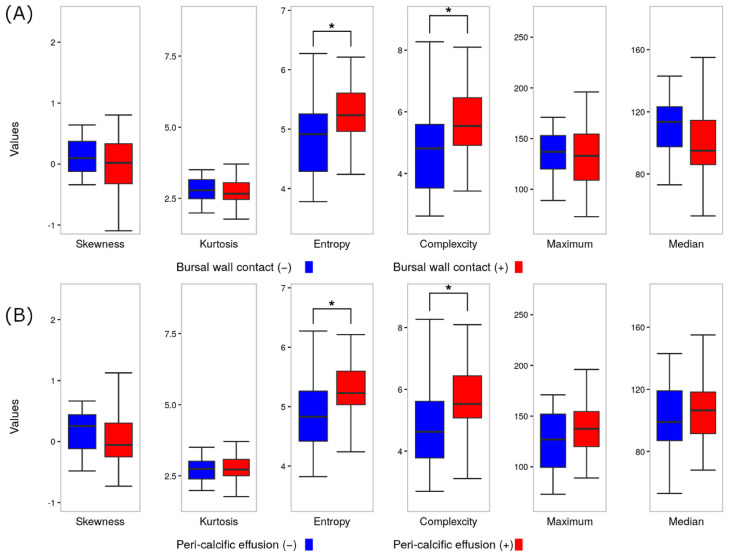
Correlations between grayscale parameters and ultrasound parameters. (**A**) Bursal wall contact and (**B**) peri-calcific effusion. * *p* < 0.05.

A focused analysis on 51 cases, which omitted 6 cases without a contemporaneous shoulder ultrasound, revealed that bursal wall contact had statistical significance with entropy and complexity values (*p* = 0.025, effect size r = 0.274; *p* = 0.031, effect size r = 0.274, respectively) compared to non-contacting calcifications (Figure 6A). Similarly, peri-calcific hypoattenuation displayed significantly elevated entropy and complexity values (*p* = 0.047, effect size r = 0.282; *p* = 0.048, effect size r = 0.282, respectively) (Figure 6B). However, neither the acoustic shadow faintness nor Sconfienza’s classification exhibited a statistically significant correlation.

## 4. Discussion

In this study, the radiopacity and internal heterogeneity of the calcific region and adjacent rotator cuff tendon were quantified. Among the parameters, entropy and complexity demonstrated the highest AUC values, representing the most distinct radiographic characteristics. Correlation analysis between the ultrasonographic and radiographic parameters revealed that ultrasound findings of peri-calcific effusion and bursal wall contact were associated with the heterogeneity parameters, while simple descriptive grayscale parameters and Gartner’s classification were not.

### 4.1. The Significance of Quantitative Analysis

Although other imaging modalities can be utilized [5], plain radiography remains the primary diagnostic tool due to its simplicity and widespread availability. Since the early 1900s, conventional radiographs have been employed to detect these radiopaque lesions [20,21]. Beyond mere detection, several qualitative classifications have been developed to characterize their dynamic progression over time. For instance, the De Palma and Gartner classifications are commonly used to categorize deposit morphology on radiographs [22,23]. Gartner described them as having a soft contour with a translucent and cloudy appearance, whereas De Palma characterized them as fluffy and amorphous.

While these qualitative classification methods demonstrate some predictive capability, they have been reported to exhibit low inter-rater reliability and a weak correlation with clinical symptoms [11,24]. In a study by Cho et al., 24% of type II calcifications reverted to type I at the final follow-up among pain-provoking calcifications successfully treated with conservative methods [25]. Given that calcifications in the resorptive phase do not realistically regress to the formative phase, this suggests discrepancies in grading among evaluators and inconsistencies between qualitative classifications and characteristics of calcification. Therefore, by quantitatively measuring radiodensity along with heterogeneous characteristics, we could potentially identify radio-morphological attributes that provide insights into the disease. Heterogeneity analysis utilizing grayscale histograms has been introduced as a valuable quantitative and objective approach to mitigate physician bias and enhance differential diagnosis across a spectrum of medical conditions, encompassing tumors, thyroid nodules, and tendinopathies [14,15,18]. This technique facilitates the measurement and quantification of various medical images, including X-ray, ultrasound, CT, and MRI, without complex computational processes [14]. Notably, a previous study leveraging grayscale histogram analysis demonstrated excellent reliability and sensitivity in detecting muscular disease [26], while another study highlighted the diagnostic utility and reliability in assessing rotator cuff tendinopathy using ultrasound images [18]. Such an approach could be necessary considering the constraints of the qualitative classification systems currently in use.

Through our findings, we aimed to address the inherent biases present in traditional qualitative classifications of shoulder radiographs, which could offer advantages for novice practitioners and in resource-constrained primary healthcare settings where CT or MRI scans are not readily accessible. Additionally, by predicting pathological conditions such as synovial irritation of the bursa and peri-calcific hypervascularization, clinicians can better anticipate detailed scenarios associated with pain provocation and impingement. In therapeutic decision-making, it is advisable to prioritize anti-inflammatory treatments over extracorporeal shock wave therapy (ESWT) for managing severe peri-calcific tendon inflammation, particularly when characterized by significant heterogeneity. This is due to the potential for short-term symptom exacerbation following ESWT and the risk of acute inflammatory responses that may intensify treatment-related discomfort [27,28]. Furthermore, precise syringe handling would be required during barbotage procedures and bursal injections to address the possibility of imminent bursal space migration and to optimize the removal of carbon apatite. Nevertheless, due to the retrospective and exploratory nature of this study with limited observed clinical variables, further clinical values will be pursued through a longitudinal observational study. This could assess changes in shoulder function scores and pain relative to the patient’s calcific phase progression.

### 4.2. Grayscale Parameters

Initially, in the descriptive statistical parameters of the grayscale values, calcifications exhibited significantly higher values across all parameters, except for the minimum values when compared to the adjacent tendon. This discrepancy can be attributed to the radiodensity differences between tendon tissue and the carbonated apatite embedded within it. Notably, the maximum grayscale values were higher by 36.97 units, and the mean values were elevated by 21.69 units.

Secondly, among the heterogeneity parameters related to asymmetric distribution, such as skewness and kurtosis, both normal tendons and calcifications demonstrated nearly symmetric and leptokurtic distributions. The median grayscale histogram values closely aligned with the mean values, both exhibiting a kurtosis of approximately 3. Notably, the heterogeneity parameters associated with disordered distribution, such as entropy and complexity, were found to be elevated in the calcifications. Previous research has shown that complexity derived from entropy increases as a distribution becomes more disordered [29]. This phenomenon can be attributed to the heterogeneous aggregation of apatite within calcific deposits, resulting from the randomness in fibrocartilaginous metaplasia, mineralization, and subsequent endocytosis. The histogram of calcific deposits revealed irregularly high-frequency bins dispersed across multiple regions, with the remainder of the distribution also being quite spread out. Consequently, the overall shape becomes less predictable, leading to higher observed entropy (Figure 3). These findings suggest that these metrics could serve as valuable indicators of the inherent structural heterogeneity in calcifications.

Tendons are highly organized tissues responsible for transmitting muscle-generated force to bones. Consequently, the orientation of tendons is influenced by the direction of force [30], resulting in a distinguishable striped structure on radiographs, such as the Achilles tendon on lateral radiographs [31]. Comprised of densely packed fascicles surrounded by low-density connective tissue [32], tendons have long been confirmed to possess a cylindrically symmetric fascicle structure through X-ray diffraction analysis [33]. Therefore, it can be speculated that tendons possess unique and organized radio-morphological features.

However, in the case of calcific deposits, no particular mechanism or observation has been identified that produces a unique radiological pattern; instead, the patterns observed tend to be random or mixed histopathology [7,34,35]. During the formative phase, irregularly rectangular crystals are aggregates in a matrix of amorphous or irregularly fragmented collagen fibers, occasionally within membrane-bound structures and infrequently embedded between collagen fibers [7]. At this stage, intraoperative examination reveals these calcific deposits as sandy, tough masses [36]. As the condition progresses to the resorptive phase, histopathological examination of the deposits shows areas of chondroid and osseous metaplasia intermixed with tendinous tissue [34]. Concurrent changes in the surrounding tendon tissues include the development of vascular channels adjacent to calcific deposits and infiltration by macrophages and multinucleated giant cells. Within these cells, phagocytosed intracellular crystalline particles are readily observable and exhibit a distinct appearance compared to extracellular deposits [7]. During certain intraoperative examinations, calcific deposits appear as toothpaste-like fluid consistency [36].

Ultimately, well-organized tendons and randomly aggregated calcific deposits exhibit markedly different levels of heterogeneity in their inner structures; thus, heterogeneity parameters could be the unique radiographic nature possessing most discriminators between these two structures. Among them, regarding the AUC, the entropy and complexity could be the most superior discriminative parameters, showing a value exceeding 0.90.

In subsequent research, it would be possible to differentiate between various radiopaque or heterogeneous ossification conditions affecting tendons, such as enthesophytes, calcific metaplasia, or the presence of foreign materials. This differentiation could be achieved by identifying characteristic grayscale values associated with these conditions. Furthermore, these radio-morphological features can be integrated into a deep learning model as feature maps within convolutional neural networks to enhance the classification of tendon diseases. A prior study by Grauhan et al. demonstrated high diagnostic accuracy in detecting shoulder osteoarthritis and dislocation, with AUCs of 0.945 and 0.896, respectively; however, the accuracy for identifying calcifications was comparatively lower, with an AUC of 0.80 [37]. Future research should leverage the high discriminative potential of heterogeneity parameters to develop more accurate classification models.

### 4.3. Ultrasonography Parameters

In the subgroup analysis comparing calcifications, we aimed to determine whether differences in grayscale parameters among calcifications are influenced by key ultrasonographic parameters, including Sconfienza’s classification, shadow faintness, bursal wall contact, and peri-calcific hypoattenuation. We observed that pathological features such as bursal wall contact and peri-calcific hypoattenuation identified by ultrasound correlate with heterogeneity measures—a relationship not previously explored in any other study. These correlations are not explained by Gartner’s classifications or simple descriptive grayscale parameters.

During the resorptive phase, calcium deposits trigger tissue inflammation and neovascularization, leading to peri-calcific effusion [7]. This process results in pain mediated by inflammatory cells [3,7]. Consequently, the affected area often softens, enlarges, and undergoes liquefaction, with the potential to extend to the bursa or migrate beyond it and finally reducing [38]. The synovium is among the most pain-sensitive tissues [39,40], and the contact of calcific deposits with the bursae can act as a primary trigger for pain. In this study, the detection of these pathophysiological changes in calcifications and adjacent tissues was accomplished through radiographic heterogeneity analysis—a capability not achievable with previous qualitative methods. This approach could be instrumental in identifying specific situations that provoke pain.

In contrast, Sconfienza’s classification, which describes the attenuation of internal calcific deposits and the faintness of shadows, showed no correlation with the grayscale parameters. The findings align with earlier reports showing no correlation with poorly defined calcifications observed in plain radiographs [13,41]. A possible explanation for this lack of correlation is that, even if variations in internal ultrasound attenuation occur, the total amount of deposited calcium may remain relatively constant, resulting in only minimal changes in radiolucency. It is possible that enough time has not yet elapsed for the pain-provoking resorptive phase calcium deposits to significantly alter radiolucency.

**Figure 7:**
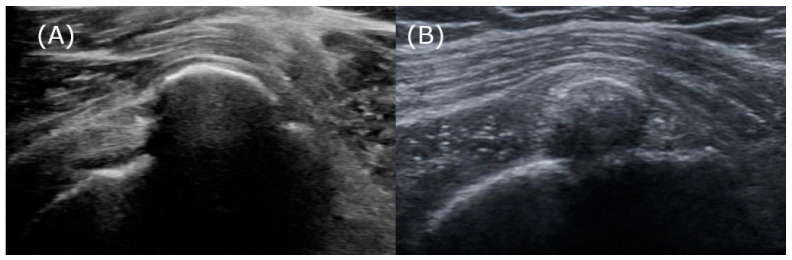
The resorptive phase calcifications, which exhibit arc-shaped attenuation and acoustic shadows beneath the bursal wall. (**A**) A 49-year-old female patient demonstrates calcification with pronounced arc-shaped attenuation and clear acoustic shadows. (**B**) A 40-year-old female patient exhibits arc-shaped attenuation with partial ultrasound penetration, allowing limited observation of the inner structure’s upper portion. The acoustic shadow is partially faint, providing a faint view of the humeral cortex.

Another plausible explanation, beyond the inaccuracies inherent in qualitative radiographic analysis, is that ultrasound attenuation is not solely determined by the stage of calcification but may also vary due to physical interactions with neighboring structures. Specifically, in confirmed cases of the resorption phase, where calcifications have been successfully removed via barbotage, the calcifications exhibit arc-shaped attenuation and produce acoustic shadows (Figure 7). The authors observed a commonality among these cases: these calcific deposits were densely packed within a confined space limited by the surrounding tendons and bursal wall. Such densely packed deposits could create significant density differences that reflect ultrasonic waves, resulting in arc-shaped attenuation from above and accompanying acoustic shadows. Therefore, it seems that relying solely on attenuation to determine the stages of calcification appears to have its limitations.

Previous studies have highlighted the drawbacks of relying on qualitative classification systems in radiological examinations. They pointed out that calcification in its formative phase might be incorrectly categorized as a resorptive phase [13,41]. These studies have exclusively emphasized the utility of ultrasound in identifying calcification stages and making informed treatment decisions. However, in the opposite cases where ultrasound cannot adequately penetrate the upper surface of calcifications, lesions in the resorptive phase may be misclassified as those in the formative phase. Notably, there is a paucity of literature documenting such misclassifications. However, in a retrospective study of 462 patients who underwent barbotage, 162 cases were designated as “hard calcifications”, characterized by a hyperechoic rim and a strong posterior acoustic shadow [19]. Interestingly, partial retrieval of the calcific material was achieved in all of these patients, and most calcifications were successfully washed away. These findings showed the challenges associated with relying solely on ultrasound attenuation to ascertain the phase of calcification, thereby highlighting the potential risk of misclassification. Such misclassifications could result in overly conservative treatment strategies. Therefore, all clinical information—ultrasound findings, radiograph parameters, and intra-tendinous calcification locations—must be taken into account, particularly when the results are discordant. This comprehensive approach allows for a more accurate interpretation of calcific stage and guides appropriate therapeutic decisions.

Future long-term follow-up studies investigating patterns of attenuation, shadow formation, and temporal changes in radiopacity and heterogeneity from the initial formative phase to the final resorptive phase could greatly enhance our understanding. Such research may highlight the crucial role of quantitative analysis in delineating the detailed and sequential pathophysiological trajectories of calcifications, thereby advancing beyond the limitations of simple qualitative classifications.

### 4.4. Limitations

This study is subject to several limitations. Firstly, as our study is retrospective, we could not assess temporal changes in calcifications and radiographic parameters with respect to clinical features. Future prospective studies investigating these temporal changes during the phasic cycle of calcifications could provide significant advancements in research. Secondly, due to the two-dimensional nature of radiographs, the parameters obtained through histogram analysis in the selected ROIs may not accurately represent the sectional heterogeneity of the entire calcification or tendon. This limitation is inherent in all methods utilizing simple radiographs, yet our findings offer a valuable quantitative complement to traditional qualitative radiographic approaches while maintaining convenience. Future research could explore histogram analyses using three-dimensional voxels through CT or MRI scans to address this limitation. Thirdly, this study predominantly included female participants, consistent with the higher prevalence of the disease in females. Future research will address this limitation by incorporating more male participants to ensure a balanced representation. Furthermore, our research focused solely on patients attending the clinic, potentially excluding individuals with asymptomatic conditions or tolerable pain levels. A forthcoming study involving a larger and more diverse patient population could help address this limitation.

## 5. Conclusions

The quantitative analysis of grayscale images revealed that calcific deposits exhibited not only increased radiopacity but also greater spatial heterogeneity, characterized by higher entropy and complexity. These features distinctly differentiate them from adjacent normal tendons. Furthermore, specific ultrasound characteristics, such as contact with the bursal wall and peri-calcific effusion, were found to influence radiographic heterogeneity. These findings may have clinical utility in predicting the pathophysiological status of calcifications and surrounding tissues.

## Figures and Tables

**Table 1:** Demographics and grayscale parameters by calcific deposit locations.

	Subscapularis	Supraspinatus	*p*-Value
	(N = 17)	(N = 40)	
Demographics			
Sex			0.667
Female	12 (70.59%)	32 (80.00%)	
Male	5 (29.41%)	8 (20.00%)	
Age	54.41 ± 7.27	57.17 ± 8.62	0.252
Side			1
Left	7 (41.18%)	17 (42.50%)	
Right	10 (58.82%)	23 (57.50%)	
BMI	24.80 ± 3.93	24.60 ± 3.45	0.894
Height	161.41 ± 5.10	160.97 ± 5.61	0.848
Weight	63.42 ± 9.54	62.96 ± 6.86	0.877
Size (mm)	7.06 ± 3.97	8.77 ± 4.98	0.217
Grayscale parameters of calcifications			
Max	137.24 ± 30.76	135.90 ± 32.34	0.886
Min	82.29 ± 30.47	79.55 ± 21.59	0.7
Mean	111.74 ± 28.48	105.74 ± 22.90	0.404
First quartile	105.06 ± 28.58	98.80 ± 21.05	0.361
Median	112.59 ± 29.10	105.53 ± 23.11	0.333
Third quartile	117.88 ± 29.16	111.42 ± 24.88	0.398
Skewness	0.03 ± 0.69	0.09 ± 0.40	0.728
Kurtosis	3.02 ± 1.41	2.86 ± 0.65	0.651
Entropy	5.08 ± 0.63	5.07 ± 0.62	0.982
Complexity	5.26 ± 1.47	5.25 ± 1.39	0.983

**Table 2:** Discriminate performance of each parameter.

Grayscale Parameters	AUC	SE	Cut-off Value	Sensitivity	Specificity	95% Confidence Interval	*p*-Value
Descriptive statistics							
Max	0.830	0.039	132.5	0.93	0.614	0.754–0.905	<0.001 ***
Min	0.594	0.054	86.5	0.789	0.456	0.489–0.699	0.080
Mean	0.749	0.045	93.6	0.684	0.702	0.66–0.839	<0.001 ***
First quartile	0.71	0.048	85.5	0.579	0.754	0.616–0.805	<0.001 ***
Median	0.747	0.046	88.5	0.579	0.807	0.658–0.837	<0.001 ***
Third quartile	0.777	0.043	97.5	0.737	0.719	0.692–0.862	<0.001 ***
Spatial heterogeneity							
Skewness	0.491	0.056	0.266	0.895	0.316	0.38–0.601	0.872
Kurtosis	0.354	0.052	5.211	1	0.035	0.251–0.456	0.005 **
Entropy	0.928	0.023	4.429	0.877	0.842	0.883–0.973	<0.001 ***
Complexity	0.928	0.023	3.798	0.877	0.842	0.883–0.973	<0.001 ***

** *p* < 0.01 and *** *p* < 0.001. Entropy was defined as −sum(Px × log2Px), where Px stands for the probability of each grayscale in the standardized histogram. Complexity was defined as E(E − 1)/4, where E is entropy.

## Data Availability

The data supporting this study are available upon request from the corresponding author. Please note that the data are not publicly accessible due to ethical considerations.
